# Assessment of Soil Moisture Anomaly Sensitivity to Detect Drought Spatio-Temporal Variability in Romania

**DOI:** 10.3390/s21248371

**Published:** 2021-12-15

**Authors:** Irina Ontel, Anisoara Irimescu, George Boldeanu, Denis Mihailescu, Claudiu-Valeriu Angearu, Argentina Nertan, Vasile Craciunescu, Stefan Negreanu

**Affiliations:** 1 Remote Sensing and Satellite Meteorology, National Meteorological Administration, 013686 Bucharest, Romania; irina.ontel@meteoromania.ro (I.O.); george.boldeanu@meteoromania.ro (G.B.); denis.mihailescu@meteoromania.ro (D.M.); claudiu.angearu@meteoromania.ro (C.-V.A.); argentina.nertan@meteoromania.ro (A.N.); vasile.craciunescu@meteoromania.ro (V.C.); 2 Department of Geography, Faculty of Sciences, University of Craiova, 200764 Craiova, Romania; giurgiustefan@yahoo.com

**Keywords:** soil moisture anomaly, SPI, LST anomaly, NDVI anomaly, drought, Romania

## Abstract

This paper will assess the sensitivity of soil moisture anomaly (SMA) obtained from the Soil water index (SWI) product Metop ASCAT, to identify drought in Romania. The SWI data were converted from relative values (%) to absolute values (m^3^ m^−3^) using the soil porosity method. The conversion results (SM) were validated using soil moisture in situ measurements from ISMN at 5 cm depths (2015–2020). The SMA was computed based on a 10 day SWI product, between 2007 and 2020. The analysis was performed for the depths of 5 cm (near surface), 40 cm (sub surface), and 100 cm (root zone). The standardized precipitation index (SPI), land surface temperature anomaly (LST anomaly), and normalized difference vegetation index anomaly (NDVI anomaly) were computed in order to compare the extent and intensity of drought events. The best correlations between SM and in situ measurements are for the stations located in the Getic Plateau (Bacles (r = 0.797) and Slatina (r = 0.672)), in the Western Plain (Oradea (r = 0.693)), and in the Moldavian Plateau (Iasi (r = 0.608)). The RMSE were between 0.05 and 0.184. Furthermore, the correlations between the SMA and SPI, the LST anomaly, and the NDVI anomaly were significantly registered in the second half of the warm season (July–September). Due to the predominantly agricultural use of the land, the results can be useful for the management of water resources and irrigation in regions frequently affected by drought.

## 1. Introduction

Drought is a complex phenomenon without a universal definition, being characterized by its implications in various fields, such as social, economic, and environmental [1]. The first visible negative effects of the drought are agricultural production [2] and natural ecosystems [3,4,5]. Agricultural drought, as defined by Wilhite and Glantz (1985), represents the decrease in soil moisture to a level that affects agricultural crops at different stages of crop development [6]. However, this decrease in soil moisture has a negative effect not only on agricultural vegetation but also on natural vegetation [7,8]. The increase in water demand due to climate change (decreasing precipitation and increasing temperatures) can lead to significant losses of biodiversity and ecosystem service [9]. Therefore, soil moisture is an essential climate variable (ECV) [10] of the land system [11,12].

In recent decades, drought events have increased in the South, Western, South-Eastern, and Central regions of Europe, while in the North and North-Eastern regions it has been decreasing [13,14,15]. The investigations of Spinoni et al. (2017) showed a significant increase in drought frequency in spring and summer, especially in the southern and western parts, and a decrease in autumn and winter in Northern and Eastern Europe [16]. The annual economic losses caused by drought are estimated in the PESETA IV project at approximately EUR 9 billion, for the EU and UK, and the highest losses being registered in Spain, Italy, and France [17].

Previous studies, based on the analysis of vegetation indices (derived from satellite data, for the period 2001–2020) have shown the frequency of spring drought in the first 10 years of the analyzed interval, while, in the last 10 years, this phenomenon occurred mainly in autumn [18]. At the regional level, the southern and eastern part of the country with increasing drought conditions is noticeable, while the northern part is highlighted by a tendency to increase humidity [19]. Regarding the frequency of the phenomenon, it is higher in the South and East [18].

Romania’s main economic sector affected by the drought conditions is agriculture. According to the inception report for the global water partnership (GWP) in Central and Eastern Europe (CEE), part of the world meteorological organization (WMO)/GWP integrated drought management program, about 14.7 million ha are frequently affected by drought in Romania [20]. In the last two decades, the largest agricultural areas affected were in 2007 (11.2 million ha), 2012 (10.3 million ha), and 2003 (9.6 million ha), followed by 2001, 2019, and 2020 [21]. The most affected areas are those in the South, Southeast, and East [18,22].

In most studies of drought, events were analyzed based on meteorological data, such as the precipitation, by calculating the standardized precipitation index (SPI) [23]; and precipitation and evapotranspiration, by calculating the standardized precipitation evapotranspiration index (SPEI) [24] or palmer drought severity index (PDSI) [25].

Furthermore, many studies are using remote sensing data in the optical, thermal, and radar field for drought assessment, including the normalized difference drought index (NDDI) [26], drought severity index (DSI) [27], vegetation temperature condition index (VTCI) [28,29], soil water index (SWI) [2], and soil moisture [30] from the European Space Agency Climate Change Initiative (ESA CCI) [31].

This study aims to analyze the quality of soil moisture anomalies obtained using the Metop ASCAT satellite to evaluate the spatio-temporal variability of drought events in Romania. The SMA was obtained based on SWI products at 3 essential depths: 5 cm (near surface), 40 cm (sub surface) and 100 cm (root zone). The results of this study can be used for the management of water resources available in agriculture.

## 2. Materials and Methods

### 2.1. Study Area

**Figure 1:**
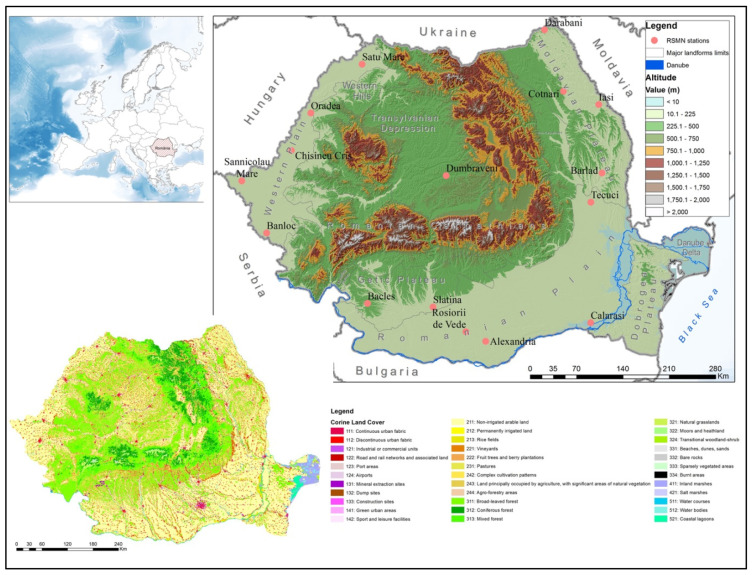
The physical-geographical characteristics of Romania and the location of the RSMN stations used in the validation of the results of the conversion of soil moisture values.

Romania has approximately 238,500 km^2^, with major relief forms distributed concentrically [32]. The Transylvanian Depression is in the center and surrounded by mountains (the Carpathian) and hills. The plains are located outside the base of the Carpathian arc (Figure 1). Most of the country is used for agriculture. According to the National Institute of Statistics [33], the agricultural area occupies 61.8% of the country’s surface. Thus, cereal crops predominate in the plain regions; in the hilly regions, the orchard and vine cultures are dominant; and, in the mountainous areas, the alpine pastures are dominant. Southern and Southeastern Romania often faces severe drought phenomena that mainly affect agricultural crops [21,34]. The sandy soils from the South–West of the Romanian Plain; the degradation of the irrigation system, after the communist period [35,36]; and the deforestation (that causes the decrease in water retention and the increase in soil scattering [37]), leads to an accentuation of the drought phenomenon in the region.

According to the Köppen–Geiger climate map [38], most of the country has a temperate-continental climate, with warm summers and no dry season (Dfb). The South and Southwest of the Romanian Plain has a temperate-continental climate, with hot summer climate and no dry season (Dfa). In the Dobrogea Plateau and the Danube Delta, there is a cold semi-arid climate with a hot and dry summer and a cold winter (BSk).

The annual mean air temperature is between −2.4 °C, in the mountainous areas, and 13 °C in the plain areas (1981–2010), and the precipitation levels are between 229.3 mm and 1649.9 mm (1981–2010) [39]. The highest temperatures are recorded in the South and East of the country (the Romanian Plain, the Dobrogea Plateau, and the Danube Delta). Moreover, these regions have the lowest amounts of precipitation. The highest amounts of precipitation are recorded in the mountainous area.

### 2.2. Data and Methodology

#### 2.2.1. Satellite Soil Moisture Products

SWI is an average product computed at a 10 day interval, 0.1° spatial resolution. It is freely provided by EUMETSAT H SAF through the program Copernicus Global Land Service (Https://land.copernicus.eu/global/products/swi, accessed on 15 June 2021), for the 2007–2020 time period [40,41]. In this regard, the 10 day average of the SWI product was analyzed. It is considered that the vegetation responds to the lack or excess of moisture within this time interval. The percentile calculation was performed based on all existing data (10 day averages). Thus, the areas with the lowest moisture (10 day synthesis) are highlighted. This product is available for various depths (5 cm, 10 cm, 15 cm, 20 cm, 30 cm, 40 cm, 60 cm, and 100 cm).

Starting from a 5 cm layer depth, it was modeled for all the above-mentioned depths, according to the product user manual [42]. The SWI is expressed in relative units (%). In this study we analyzed satellite products for depths of 5 cm, 40 cm, and 100 cm, and validation was performed only for data from 5 cm depths, which match the depth of the in situ measurements. The conversion into volumetric units (m^3^/m^3^) has been performed, in order to analyze the spatial distribution over Romania of soil moisture in volumetric units, and also to validate it with in situ measurements.

Therefore, the soil bulk density ($\rho_{b}$) and mean density of soil particles ($\rho_{s}$) for soil moisture (SM) conversion have been used, as presented in Equation (1) [43]:

(1) $$\mathrm{SM}=\mathrm{SWI}\left[\left(1-\frac{\rho_{b}}{\rho_{s}}\right)-\theta_{r}\right]+\theta_{r}$$

where $\rho_{s}$ is the mean density of the soil particles (constant value 2.65 g/cm^3^) [44,45]; $\theta_{r}$ is the residual SM being calculated as the average of the minimum SM values recorded at the in situ stations, and the resulting value was 0.04 m^3^/m^3^; and $\rho_{b}$ is the soil bulk density (fine earth) 10 × kg/m^3^. The source of bulk density data used is provided by Hengl, 2008 [46]. Thus, considering that soil bulk density values are available for the depths of 0, 10, 30, 60, 100, and 200 cm, and the depths for the computed SWI products are 5, 40, and 100 cm depths, the average of 2 consecutive layers of soil bulk density was used (Figures S1–S3). The quality of the transformation result depended on the additional input data [47].

#### 2.2.2. In Situ Soil Moisture

The in situ soil moisture datasets (5 cm depth) from the Romanian Soil Moisture Network (RSMN) [48], which are part of the International Soil Moisture Network (ISMN) [49,50], were used to validate the SM results. The data is freely available through the ISMN (https://ismn.geo.tuwien.ac.at/en/, accessed on 30 June 2021). The sensor used in the RSMN network is 5TM. It measures the dielectric permittivity at a frequency of 70 MHz, which minimizes the influences due to soil texture and salinity. The time interval between the soil moisture measurements is 60 min at a 5 cm depth. Moreover, the soil moisture observations are quality controlled by the National Meteorological Administration (NMA). In order to be able to compare them with the soil moisture in the SWI product, they were mediated at 10 day intervals. The differences between soil moisture from remote sensing and in situ were quantified by the standard statistical methods. Thus, the bias, median absolute error (MAE), root mean square deviation (RMSD), the Pearson correlation coefficient (r), and the Spearman’s rank correlation coefficient (rho) were calculated.

There are 4 soil moisture stations in the Romanian Plain, 5 stations in the Western Plain, 1 in the Transylvanian Depression, 4 in the Moldavian Plateau, and 2 in the Getic Plateau, covering a period of at least 5 years of registration. The characteristics of the RSMN dataset used in this study are summarized in Table 1.

The identification of drought events was based on the SMA using a z-score. This indicator was computed based on the SWI 10 day products. We calculated the average (*µ*), standard deviation (*σ*), and finally the anomaly (*δ*), according to Equation (2). The Shapiro–Wilk normality test was applied to check if the data is distributed normally or not. All the *p* values are larger than 0.05, thus, the distribution of the 10 day datasets is not significantly different from the normal distribution. The results can be found in the Supplementary: Table S1 and Figure S4. The resulting values were classified into 5 categories (3–for drought, 1–normal condition, and 1–wetter than normal), according to Table 2:

(2) $$\delta =\frac{x-µ}{\sigma }$$

where: *x*—is the 10 days period average; *µ*—is the average for the period 2007–2020; and *σ*—is the standard deviation, which is calculated for the same period (2007–2020).

The SMA was validated by several drought indices, including the NDVI anomaly and LST anomaly. Both the NDVI and LST were resampled to 0.1° for each 10 day period, between 2007 and 2020. The data used for the NDVI and LST anomaly were provided by the Terra-MODIS, MOD09GA product [52] and MOD11A1 product [53], respectively. Moreover, both indices are processed and downloaded using the Google Earth Engine (GEE) platform.

NDVI is a well-known vegetation index [54]. It is frequently used in studies aimed at analyzing the impact of drought on vegetation, the results being satisfactory [55,56]. The negative values of the NDVI anomaly (less than −1) represent browning vegetation, when they are between −1 and 1 they represent the vegetation in normal vegetation conditions, and when they are over 1 they represent greening vegetation.

LST is an essential climate variable with implications in land–atmosphere exchange processes [10]. High temperatures can contribute to an increase in evapotranspiration and drought. Thus, the positive values (above 1) of the LST anomaly often highlight drought occurrence due to thermal stress [57]. Moreover, there is a close correlation between the browning of vegetation resulting from NDVI, and the increase in temperature stress highlighted by LST [58,59,60,61].

#### 2.2.3. Drought Indicators from Meteorological Data

According to the World Meteorological Organization (WMO), SPI is a key meteorological drought indicator, which is computed using the amount of precipitation that is typically accumulated at the intervals of 1, 3, 6, 9, 12, 24, or 48 months [62]. In this study, the SPI at 10 day intervals is calculated between 2007 and 2020, relative to the 1961–2020 reference period. The SPI classification is similar to that of the SMA, in Table 2. By using data registered at the meteorological stations, raster, with a resolution of 0.1°, were obtained. The data used are part of the archive of the National Meteorological Administration (NMA).

## 3. Results

### 3.1. Soil Moisture Conversion Accuracy

**Figure 2:**
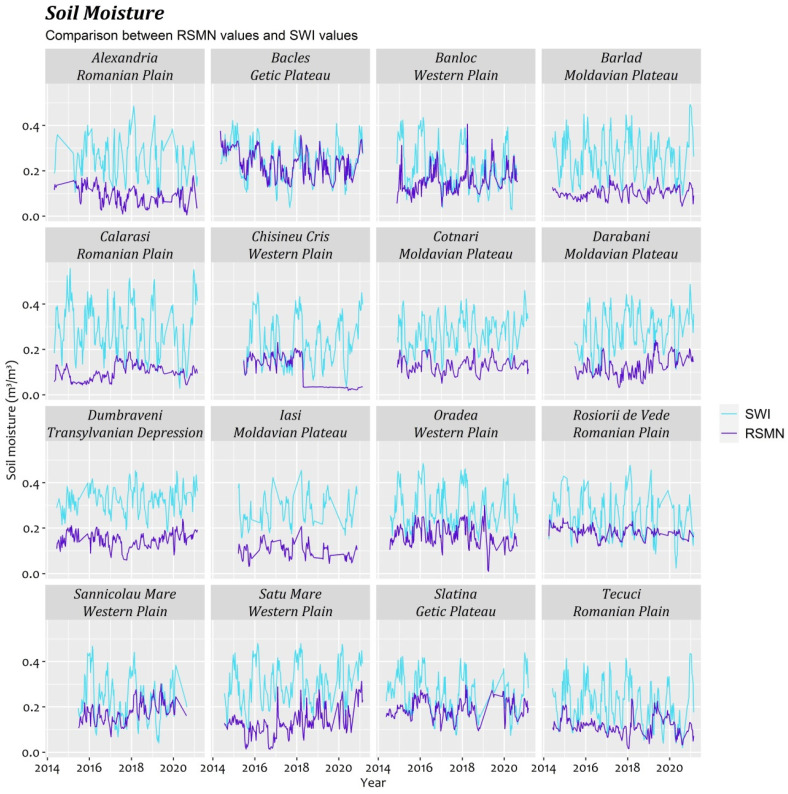
Comparison between soil moisture from SWI with in situ (RSMN) at a 5 cm depth.

Figure 2 presents the two data sets (remote sensing vs. in situ) for the period 2015–2020. We noted that, generally, satellite data overestimated the soil moisture. The estimated values of SM are higher than the in situ data at 10 of the 16 stations analyzed. However, there are some stations in which the correlation is satisfactory (Bacles, Banloc, Slatina, and Sannicolau Mare).

The correlation coefficients over 0.6 were obtained for the stations located in the Getic Plateau (Bacles (r = 0.797 and rho = 0.809) and Slatina (r = 0.672 and rho = 0.708)), in the Western Plain (Oradea (r = 0.693 and rho = 0.724)), and in the Moldavian Plateau (Iasi (r = 0.608 and rho = 0.591)), in Table 3. The average of the significant Pearson correlation coefficients (r) was 0.491 and Spearman (rho) was 0.51, while greater than 0.5 were at 8 out of 16 stations. However, the bias values are small, being between −0.009 at the Bacles station (located in a hill area at 313.0 m alt., being the second station with the highest altitude) and −0.186 at the Calarasi station (located in the low meadow area at 18 m alt., having the lowest altitude of all the analyzed stations). Overall, the mean value of bias per RSMN network was −0.122.

The RMSE and MAE values are between 0.05 and 0.184, and between 0.04 and 0.178, respectively. The lowest values were registered at the Bacles and Slatina stations, and the highest at Calarasi. The mean value of RMSE per RSMN network was 0.148 and MAE was 0.130.

There is a connection between the correlation coefficients and the percentage of sandy soil (r = 0.65) and the percentage of clay soil (r = −0.56). It was observed that where the sand percentage in the soil is higher and the clay percentage in the soil is lower, the correlation between the satellite and in situ data is better. Moreover, it was observed that the correlation is better at the stations located at higher altitudes than those located at lower altitudes, with r = 0.53. Furthermore, no connection was identified between these differences or the distribution of the stations by major landforms, or by latitude and longitude.

### 3.2. Soil Moisture Distribution (2007–2020)

In order to better highlight the spatial distribution of SM in Romania, the values of the first quartile (Q_1_ or 25%), the second or median quartile (Q_2_ or 50%), and the third quartile (Q_3_ or 75%) were extracted. Therefore, the first quartile shows the dry areas in the country.

**Figure 3:**
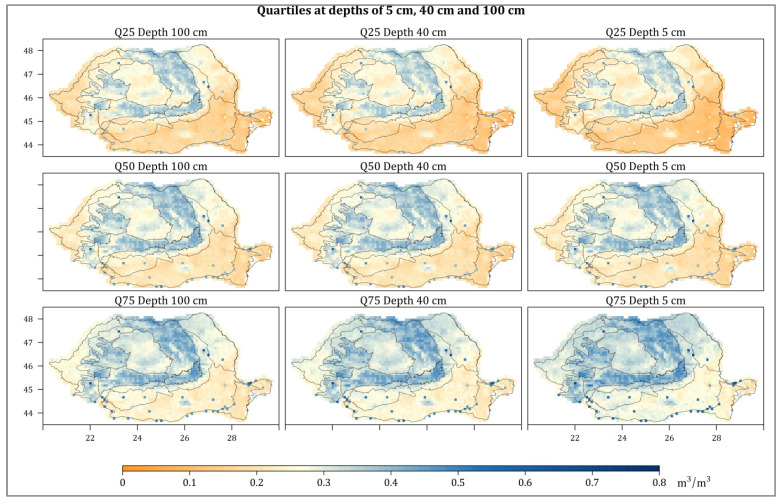
The SM quartile (Q_1_, Q_2_, and Q_3_) for 5 cm, 40 cm, and 100 cm depth in Romania (2007–2020). The quartiles were identified based on the average values of SM at intervals of 10 days.

According to the Q_1_ of SM at 5 cm depth, the lowest values of the SM are registered in the South-East of Romania, in the low meadow area and to the east of the Romanian Plain, the South of the Moldavian Plateau, the entire Dobrogea Plateau, and the Danube Delta (0.06 to 0.13 m^3^/m^3^), as is presented in Figure 3. Therefore, the southeast area can be considered as the driest in Romania, in which the soil moisture frequently registers low values (<0.13 m^3^/m^3^), hence the high risk of drought. In the South and Southwest of the Romanian Plain and in the Western Plain, the values are slightly higher (between 0.13 and 0.18 m^3^/m^3^), gradually increasing with the altitude. The MS at 40 and 100 cm depth has slightly higher values than at 5 cm depth, but the same spatial distribution is maintained at the country level. Q_2_ and Q_3_ highlight the same spatial distribution of SM in Romania as Q_1_. However, the values are approximately 0.1 m^3^/m^3^ and higher. The distribution of soil moisture in Romania corresponds to the type of climate in each area [63].

At a monthly level, the mean values of SM at 5 cm depth in the period 2007–2020 in the Romanian Plain, was about 0.18 m^3^/m^3^ in the April–May period, and slightly higher (0.22 m^3^/m^3^), in September–November. Generally, the values decreased from West to East. Moreover, the low values of SM in April–May were recorded in the Dobrogea Plateau (0.15 m^3^/m^3^).

At 40 cm and 100 depths, the lowest monthly average values were registered in the Romanian Plain and the Dobrogea Plateau in May–August (about 0.17 m^3^/m^3^), and the highest in November–December (about 0.21 m^3^/m^3^ in the Romania Plain and 0.19 m^3^/m^3^ in the Dobrogea Plateau). Slightly lower values were observed at 40 cm and 100 cm depths compared to 5 cm (at the surface) in the Romanian Plain, while, in the Dobrogea Plateau, the values in depth were higher than those at the surface.

### 3.3. Drought Description according to SMA in Romania

**Figure 4:**
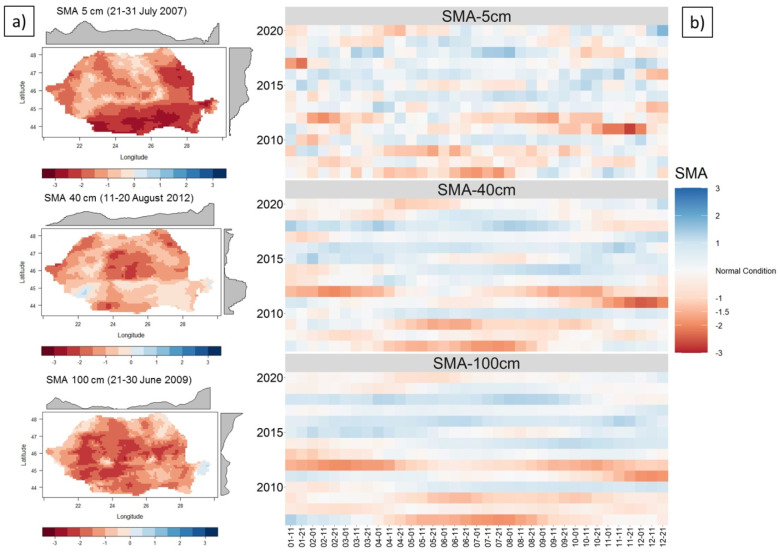
SMA in Romania at 5 cm (top), 40 cm (center), and 100 cm (bottom) depth (2007–2020): (**a**) spatial distribution of SMA from 21–31 July 2007 (top), 11–20 August 2012 (center), and 21–30 June 2009 (bottom); and (**b**) mean of SMA for 10 day intervals.

SMA was used to characterize the intensity, spatial extent, and duration of the drought event. Therefore, the extreme and severe droughts were recorded at the beginning of the analyzed period, in 2007–2009, 2011, and 2012 (Figure 4). The first driest year was 2007 and the second was 2012. Moderate drought was recorded, especially in the years after 2015 when an increase in soil moisture was observed, especially in depth (100 cm). Additionally, the dry events at 40 cm, and 100 cm depths are longer compared to those at 5 cm, where there is a greater variability of SM.

In 2007, the extreme drought affected all of Southern (Romanian Plain) and Eastern Romania (the Moldavian and Dobrogea Plateau), while, in 2012, it mainly affected the center (Transylvanian Depression), west (Western Plain), and southwest (Romanian Plain). In 2009, the extreme drought was recorded mainly in the central part of Romania, but also in small areas in the South and East of the country. Severe and moderate drought are the most frequent types of drought present in Romania. These affected large agricultural areas (over 5 million ha) in 2009, 2011, 2012, 2018, 2019, and 2020 (at 5 cm depth). Extreme drought was recorded in 2007 (almost 5.7 million ha affected between 21 and 31 July, at 5 cm depth), 2011 (7.1 million ha affected between 11 and 20 Nov, at 5 cm depth), and 2012 (5.7 million ha affected between 1 and 10 April, at 100 cm depth). The agricultural surfaces affected by drought, in the driest years, are presented in Table S2.

### 3.4. Correlation of Drought from SMA with SPI, LST, and the NDVI Anomaly

**Figure 5:**
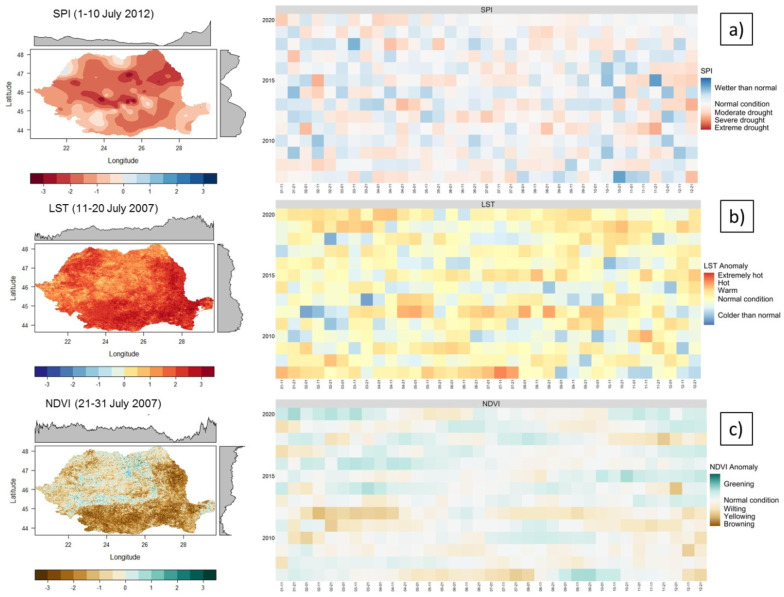
Variability of: (**a**) SPI, (**b**) LST anomaly, and (**c**) NDVI anomaly in Romania (2007–2020). The indices were calculated at 10 day intervals to be compatible with the SMA.

The SPI, LST anomaly, and NDVI anomaly were computed in order to identify the possible connection with SMA. Figure 5 presents the mean values of the three indicators in Romania (right side), and one example for each indicator with spatial extensions (left side). The values are the mean for the entire surface of Romania.

The SPI indicated normal conditions in terms of accumulated precipitation over 10 day, intervals for most of the analyzed period. However, there have been 10 day periods with moderate and severe drought over a large area of Romania (2011–2012).

The LST anomaly showed a positive anomaly of temperatures, with long warm or hot periods in 2008–2009, 2015, and 2018–2020, and extremely hot periods in 2007 and 2012. The NDVI anomaly showed a yellowing to browning of the vegetation mainly in the last two years mentioned. Furthermore, it highlighted the browning vegetation in the spring of 2012. This negative anomaly can be due to the drought at the end of 2011, which especially affected the autumn crops.

**Figure 6:**
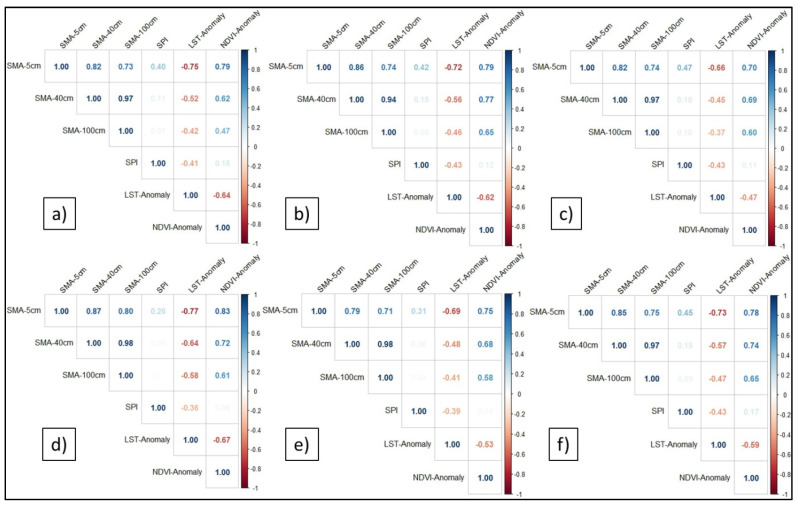
The correlations between the soil moisture anomaly (5 cm, 40 cm, and 100 cm depths) and the SPI, LST anomaly, and NDVI anomaly in the second part of the warm seasons (July–September) within: (**a**) the Romanian Plain; (**b**) Western Plain; (**c**) Transylvanian Depression; (**d**) Moldavian Plateau; (**e**) Dobrogea Plateau; and (**f**) Getic Plateau.

**Figure 7:**
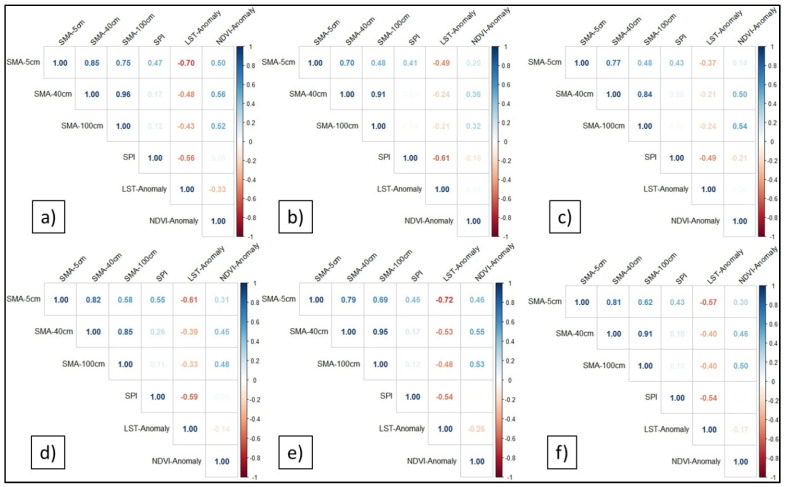
The correlations between the soil moisture anomaly (5 cm, 40 cm, and 100 cm depths) and the SPI, LST anomaly, and NDVI anomaly in the first part of the warm seasons (April–June) within: (**a**) the Romanian Plain; (**b**) Western Plain; (**c**) Transylvanian Depression; (**d**) Moldavian Plateau; (**e**) Dobrogea Plateau; and (**f**) Getic Plateau.

Finally, by using the Pearson correlation coefficient, the agreement between the drought obtained from SMA and the common indices (SPI, LST anomaly, and NDVI anomaly) has been evaluated for Romanian major landforms. Initially, the correlations were analyzed over different time intervals (annual and seasonal), but it was found that the best correlation was recorded for the July–September interval (Figure 6), followed by the April–June interval (Figure 7).

Generally, the results showed a moderate-to-strong positive correlation between the SMA and NDVI anomaly, and a moderate-to-strong negative correlation between the SMA and LST anomaly, for all the landforms in the second part of the warm seasons (July–September) (Figure 6). SMA and SPI have weaker correlations. However, these results need to be further studied by increasing the time step of the SPI from 10 days to at least 1 month. Furthermore, the correlation coefficient is higher for SMA at 5 cm and lower for 40 cm and 100 cm depths.

## 4. Discussion

Several aspects related to the soil moisture estimated from Metop ASCAT and related to drought from SMA were observed.

The estimated data form about half of the 16 in situ soil moisture stations in Romania match those measured. The issue of validating soil moisture can be due to the mismatch in spatial scales [64]. The in situ data are values measured at a single point, while satellite data are observed values over an area of 0.1°. However, we observed a good correlation at the stations located on soils with a high percentage of sand and a low percentage of clay. According to Liu et al. (2010) [65], the effect of cracking soils can occur during dry periods if the percentage of clay is high, resulting divergences between satellite data and those measured punctually at the station. Rainwater is no longer evenly distributed on the surface but accumulates in the cracks formed, so the sensor at the station can register a lower or higher value than the needles recorded by the satellite sensor.

The general results of our validation match those of the SWI product validation reports [66,67]. However, the results presented in those reports are from stations in which a good correlation was observed, such as Bacles or Oradea. The stations with poor correlation results, such as Calarasi and Alexandria, were omitted. These are located in the main agricultural areas of the country and it is important to know how accurate the soil moisture estimate is. Therefore, it was necessary to make this analysis in order to be able to go further in the analysis of the drought. However, in Romania there are a limited number of automatic stations for measuring the soil moisture. The correlations between the two data sources were made only for the locations with measurements for the available period of time (2015–2020). At the same time, the in situ sensors perform measurements at a fixed point, which is not representative of neighboring areas, compared to the satellite images that provide information at the regional or global level.

Even if there is a difference between the estimated and the measured data, the drought events identified by the SMA method match those identified by the NDVI anomaly and the LST anomaly, and less by the SPI. The short time interval (10 days) compared to the typical one for which the SPI is calculated (1, 3, 6 months, and so on), did not allow the clear identification of the periods with severe or extreme drought, already known in the literature [22,68]. Moreover, according to Sehler et al. [69], the precipitation and soil moisture have weaker correlations in temperate areas and areas with higher amounts of precipitation, compared to areas in which small amounts of precipitation are recorded. This would explain the different results from one relief unit to another, as well as from one season to another. For example, compared to the Romanian Plain, the amount of precipitation in the Western Plain is higher and drought occurs occasionally and less intensely, thus explaining the weak correlation.

Some areas of Romania, such as the Romanian and Western Plains, are frequently affected by heat waves and drought [2,70,71], and the increase in aridity in these regions is projected [72]. The main cause of drought occurrence is the high temperature that causes the decrease in soil moisture, having a negative impact on vegetation. Moreover, the drought of the last decades has led to a decrease in groundwater resources in Eastern Romania [73,74]. In Southern Romania, the air temperature exceeded 40 °C in 2007 [75] and 35 °C in 2012 [71], also obviously influencing the LST. The high temperatures combined with low rainfall mainly affected agricultural crops, causing lower production levels. The agricultural areas affected by the drought, as it results from the SMA, are close to those that resulted from our previous work [21]. However, the differences exist as a result of the differences in spatial resolution and the reference periods taken into account (2007–2020 compared to 2001–2019). Previously, the results were based mainly on optical satellite images, and, in the present paper, the results are based on SAR data. In this paper, the surfaces affected by droughts at different depths (5 cm, 40 cm, and 100 cm) have been quantified. It can be observed that there are differences in the extension of the surfaces affected by the drought at surface level (5 cm) compared to those in depth (40 cm and 100 cm).

The changes in soil moisture at 40 cm and 100 cm depths are less sensitive to the changes in precipitation compared to the surface layers [76]. Water infiltration into the lower layers of the soil depends on its texture and structure, but also on the amount and intensity of precipitation [77]. This is mainly due to the amount of precipitation that evaporates before reaching the lower layers of the soil. Additionally, wind erosion has a significant effect on drought, especially in sectors with sandy soils (Romanian Plain) [78]. Moreover, the heavy rain causes a rapid drainage of water on dry soil [79]. Compaction and crusting act mainly in the plain region in the South and West of the country, in which these processes are most widespread [78]. Therefore, even if the monthly precipitation registered at the meteorological stations does not highlight a drought event, the soil moisture in the lower layers of the soil can have values below the normal period. The occurrence of drought at the depths of 40 cm and 100 cm could have a negative impact on drought-prone plant communities [80]. Water erosion is a strong drought-amplifying factor in the Dobrogea Plateau (the main area of the country subject to desertification), as well as in the Barlad Plateau (South of the Moldavian Plateau), and in the Getic Plateau [81].

## 5. Conclusions

The results of the study led to the following conclusions:

(i) They comply with the results of the validations performed in other studies from other regions. The RMSE were between 0.05 and 0.184, and the mean correlation coefficient (r) was 0.491. The correlation between the measured data and those recorded from satellite data, represents a challenge that will be studied in detail in future studies.
(ii) The results indicated that the lowest soil moisture values are in the entire Romanian Plain and Dobrogea Plateau, Western Plain, and southern parts of the Moldavian Plateau. By knowing the areas with low soil water content values, the feature of irrigation can be improved.
(iii) SMA highlighted very well two excessively dry agricultural years (2011–2012 and 2007). They appeared in the official reports as having a recurrence period of 3 events at 10 years, respectively 1 event at 25 years [81]. Moreover, other drought events have been identified, such as those in 2009, 2019, and 2020. The results of the correlation between SMA and various drought indices (SPI, the LST anomaly, and NDVI anomaly) are confirmed by previous studies [24,82]. Thus, a moderate-to-strong correlation was observed in the July–September interval compared to the April–June interval, in which a weak correlation was observed.

Future studies should aim to test other methods of transformation from relative values (%) to absolute values (m^3^/m^3^), such as cumulative distribution function (CDF) matching [83], and add other drought indicators, such as SPEI [84], that take into account evapotranspiration to validate the results.

## Figures and Tables

**Table 1:** Characteristics of the RSMN stations used.

ID	Name	Lon	Lat	Alt (m)	Period	Soil Fraction (0–0.3 m Depth) [51]				Major Landforms
						Clay	Organic Carbon	Sand	Silt	
15489	Alexandria	25.35	43.97	75.46	2014–2020	25	1.65	28	47	Romanian Plain
15460	Calarasi	27.33	44.20	18.7	2014–2020	47	0.97	14	39	
15470	Rosiorii de Vede	24.97	44.10	102.2	2014–2020	47	1.81	19	34	
15265	Tecuci	27.40	45.84	60.0	2014–2020	18	0.6	34	48	
15289	Banloc	21.13	45.38	83.4	2014–2020	54	1.13	22	24	Western Plain
15199	Sannicolau Mare	20.60	46.07	85.0	2015–2020	18	0.6	34	48	
15136	Chisineu Cris	21.54	46.51	96.0	2015–2020	43	1.35	17	40	
15080	Oradea	21.89	47.03	136.0	2014–2020	21	0.86	39	40	
15010	Satu Mare	22.88	47.72	123.0	2014–2020	24	0.83	47	29	
15189	Dumbraveni	24.59	46.22	318.0	2014–2020	19	0.99	37	44	Transylvanian Depression
15000	Darabani	26.57	48.19	259.0	2015–2020	22	1.65	39	39	Moldavian Plateau
15056	Cotnari	26.92	47.35	289.0	2014–2020	41	1.13	27	32	
15090	Iasi	27.62	47.16	74.3	2014–2020	25	1.65	28	47	
15197	Barlad	27.64	46.23	172.0	2014–2020	25	1.65	28	47	
15434	Slatina	24.35	44.44	172.0	2014–2020	18	0.6	34	48	Getic Plateau
15412	Bacles	23.11	44.47	313.0	2014–2020	19	0.99	37	44	

**Table 2:** Drought category.

SMA Value	Drought Category
>1	Wetter than normal
−1 to 1	Normal condition
−1 to −1.5	Moderate drought
−1.5 to −2	Severe drought
≤−2	Extreme drought

**Table 3:** The correlation coefficients and the errors between SM-RSMN and SM-SWI.

Name	SM-RSMN vs. SM-SWI					Sample
	Bias	RMSE	MAE	r	rho	
Alexandria	−0.158	0.176	0.160	0.403	0.404	164
Calarasi	−0.186	0.215	0.187	0.237	0.243	219
Rosiorii de Vede	−0.078	0.110	0.093	0.515	0.526	187
Tecuci	−0.095	0.128	0.102	0.354	0.418	224
Banloc	−0.066	0.114	0.086	0.311	0.396	193
Sannicolau Mare	−0.059	0.106	0.084	0.428	0.473	158
Chisineu Cris	−0.133	0.172	0.140	0.214	0.198	186
Oradea	−0.119	0.134	0.120	0.693	0.724	202
Satu Mare	−0.167	0.183	0.167	0.564	0.577	212
Dumbraveni	−0.178	0.184	0.178	0.474	0.453	207
Darabani	−0.16	0.178	0.161	0.453	0.495	182
Cotnari	−0.146	0.158	0.146	0.542	0.556	202
Iasi	−0.176	0.184	0.176	0.608	0.591	126
Barlad	−0.157	0.175	0.157	0.586	0.586	223
Slatina	−0.069	0.094	0.077	0.672	0.708	197
Bacles	−0.009	0.050	0.040	0.797	0.809	226
Mean	−0.122	0.148	0.130	0.491	0.510	16

## Data Availability

Not applicable.

## Supplementary Materials

The following are available online at https://www.mdpi.com/article/10.3390/s21248371/s1, Figure S1: Mean of Bulk Density at 0 cm and 10 cm depth for conversion of soil moisture at 5 cm depth (data source: Tomislav Hengl. 2018). The data was processed and downloaded using the Google Earth Engine platform, Figure S2: Mean of Bulk Density at 30 cm and 60 cm depth for conversion of soil moisture at 40 cm depth (data source: Tomislav Hengl. 2018). The data was processed and downloaded using the Google Earth Engine platform, Figure S3: Bulk Density at 100 cm depth for conversion of soil moisture at 100 cm depth (data source: Tomislav Hengl. 2018). The data was processed and downloaded using the Google Earth Engine platform, Figure S4: Histogram of the 10-day dataset (1–10 June) from 2007 to 2020 for No 1 to 8 cells, Table S1: The coordinates and p-value of the cells for which the data distribution was test for normality using Shapiro-Wilk Normality Test, Table S2: Maximum agricultural surfaces (ha) affected by drought each year according to SMA (2007–2020) and 10-day intervals when they were registered.
