# Supplementary material for: Assessment of Soil Moisture Anomaly Sensitivity to Detect Drought Spatio-Temporal Variability in Romania
Source: Sensors (Basel). 2021 Dec 15;21(24):8371. doi: 10.3390/s21248371 (PMC8708061; doi:10.3390/s21248371)
Supplement: Supplementary file 1 [file sensors-21-08371-s001.zip › sensors-1437250-supplementary.pdf]

Assessment of Soil Moisture Anomaly Sensitivity to Detect Drought Spatio-Temporal Variability in Romania

Irina Ontel[1], Anisoara Irimescu[1*], George Boldeanu[1], Denis Mihailescu[1], Claudiu-Valeriu Angearu[1], Argentina Nertan[1], Vasile Craciunescu[1] and Stefan Negreanu[2]

[1] Remote Sensing and Satellite Meteorology, National Meteorological Administration, Bucharest, 013686, Romania; irina.ontel@meteoromania.ro (I.O.); anisoara.irimescu@meteoromania.ro (A.I.), george.boldeanu@meteoromania.ro (G.B.); denis.mihailescu@meteoromania.ro (D.M.); claudiu.angearu@meteoromania.ro (C.A.); argentina.nertan@meteoromania.ro (A.N.); vasile.craciunescu@meteoromania.ro (V.C.);

[2] Department of Geography, Faculty of Sciences, University of Craiova, 200764 Craiova, Romania; giurgiustefan@yahoo.com (S.N.);

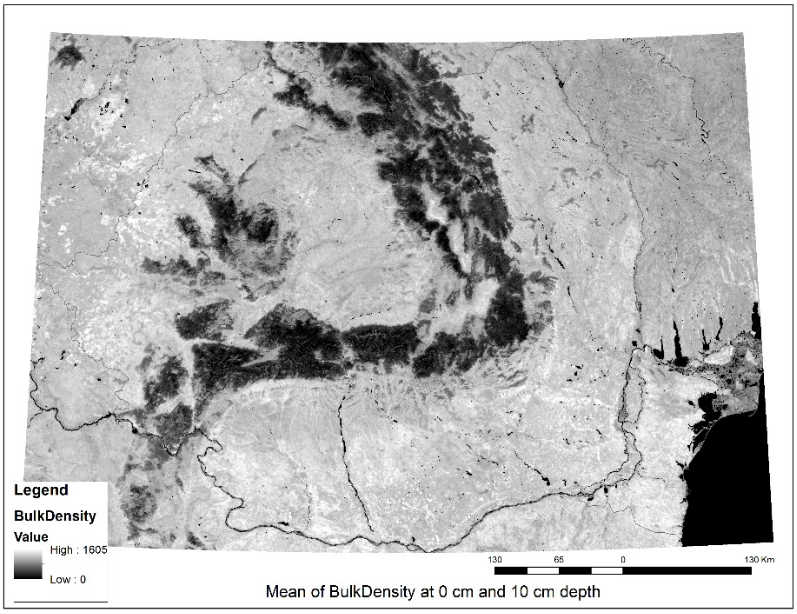

Figure S1. Mean of Bulk Density at 0 cm and 10 cm depth for conversion of soil moisture at 5 cm depth (data source: Tomislav Hengl. 2018). The data was processed and downloaded using the Google Earth Engine platform.

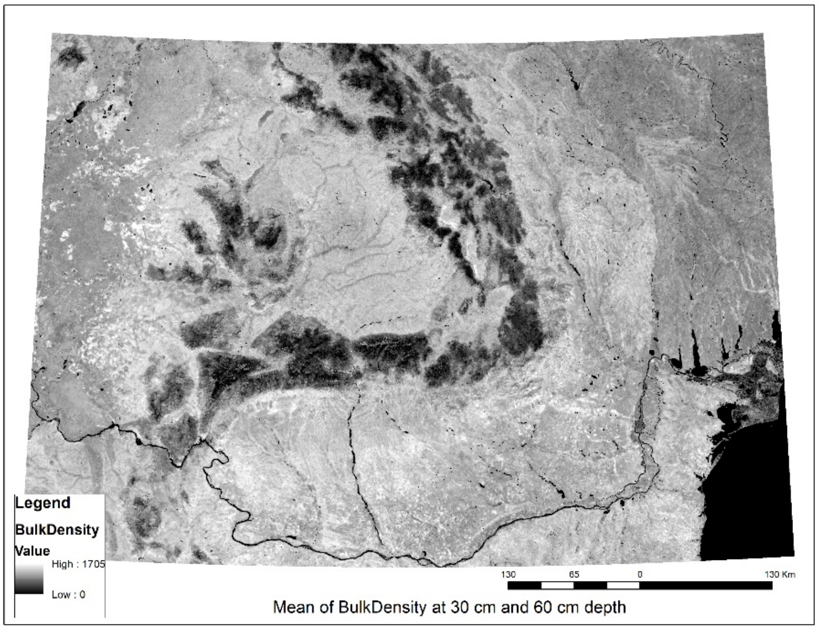

Figure S2. Mean of Bulk Density at 30 cm and 60 cm depth for conversion of soil moisture at 40 cm depth (data source: Tomislav Hengl. 2018). The data was processed and downloaded using the Google Earth Engine platform.

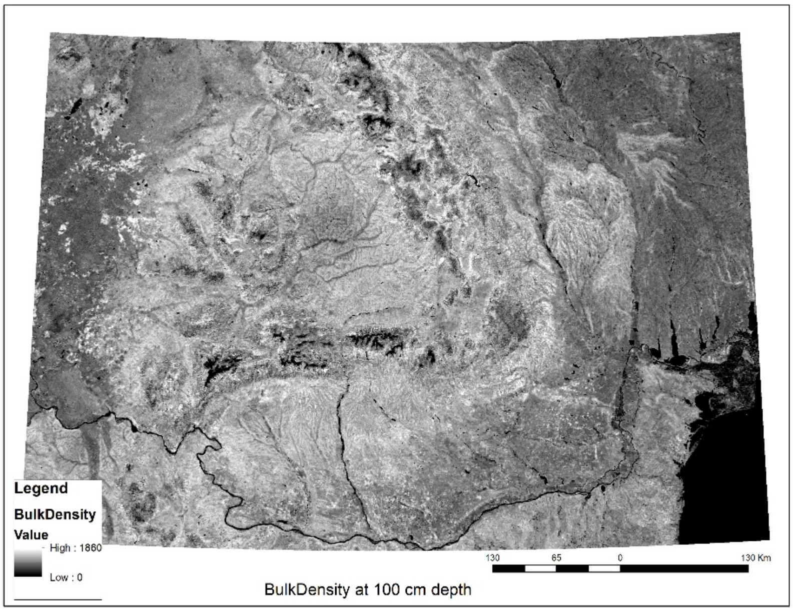

Figure S3. Bulk Density at 100 cm depth for conversion of soil moisture at 100 cm depth (data source: Tomislav Hengl. 2018). The data was processed and downloaded using the Google Earth Engine platform.

Table S1. The coordinates and p-value of the cells for which the data distribution was test for normality using Shapiro-Wilk Normality Test

No	Lat.	Long	Shapiro-Wilk Normality Test (p-value)

1	44.03167	23.274357	0.1602
2	44.08167	23.074357	0.15
3	44.98167	24.274357	0.6572
4	45.28167	26.574357	0.236
5	45.68167	21.424357	0.2363
6	46.18167	26.224357	0.6992
7	46.78167	25.524357	0.2927
8	47.58167	24.974357	0.4358

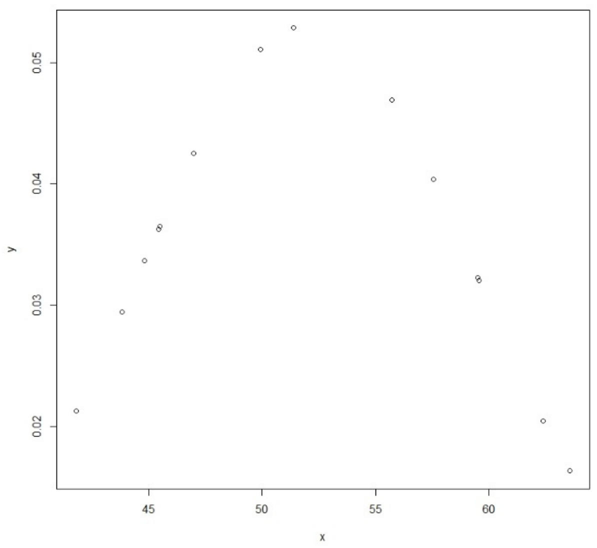

No 1

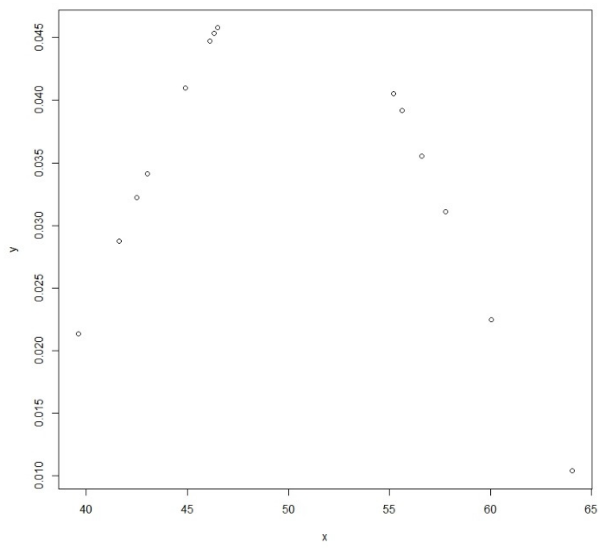

No 2

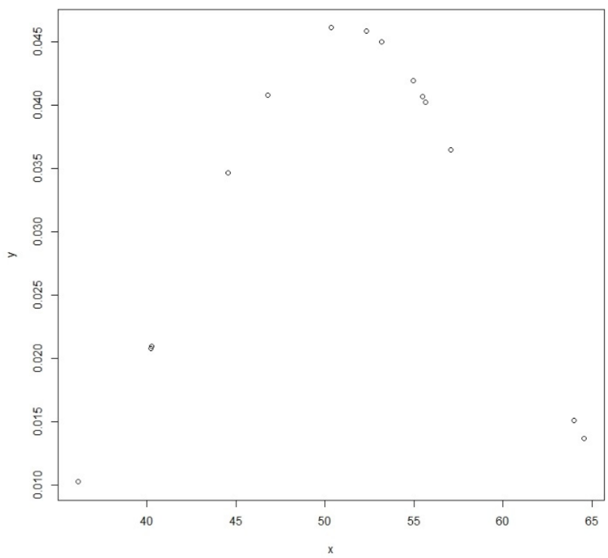

No 3

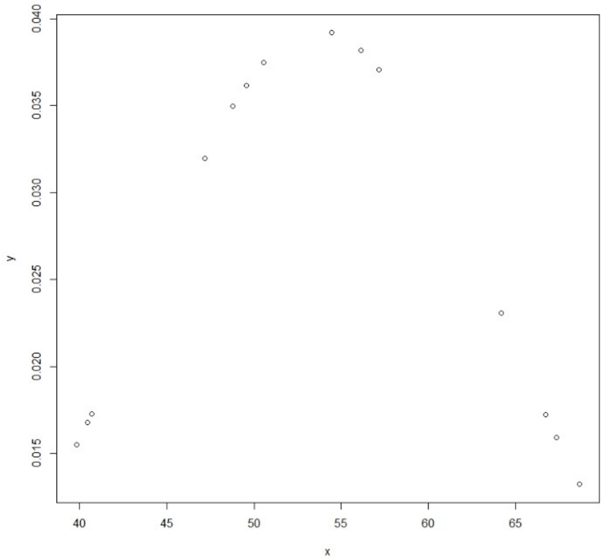

No 4

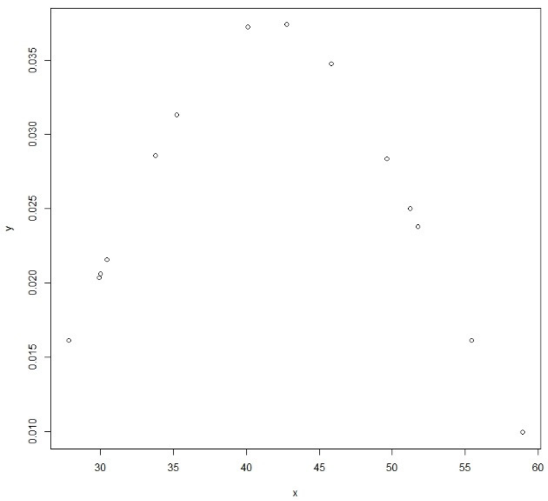

No 5

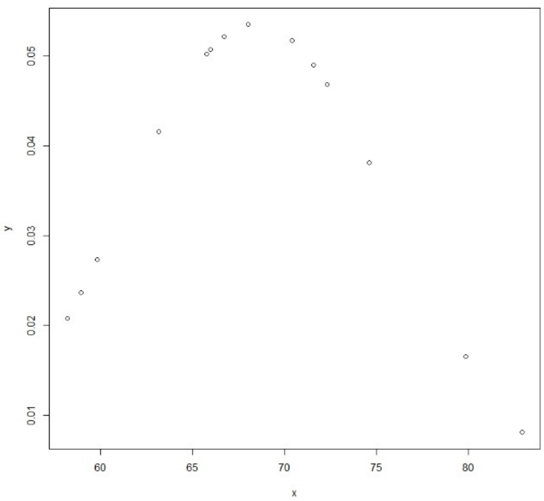

No 6

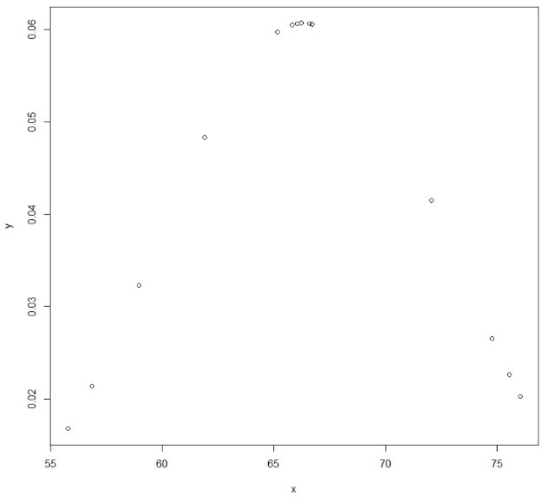

No 7

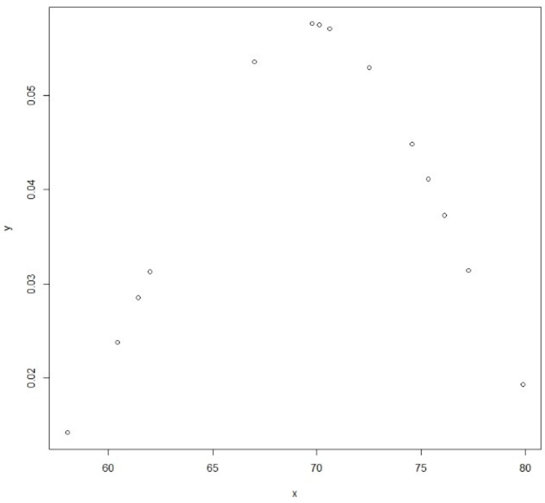

No 8

Figure S4. Histogram of the 10-day dataset (1-10 June) from 2007 to 2020 for No 1 to 8 cells.

Table S2. Maximum agricultural surfaces (ha) affected by drought each year according to SMA (2007-2020) and 10-day intervals when they were registered.

	Interval	5 cm depth			
		Moderate (ha)	Severe (ha)	Extreme (ha)	Total (ha)
2007	21-31 Jul	3158848	3649223	5678766	12486837
2008	1-10 Sep	2953315	2067705	2095299	7116319
2009	21-31 May	3918890	5596734	3343543	12859167
2010	21-31 Mar	1167445	355720	258489	1781654
2011	11-20 Nov	907211	5149213	7128046	13184469
2012	21-30 Sep	3117444	758065	1001254	4876762
2013	1-10 May	3156847	346814	2042	3505703
2014	1-10 Apr	1378519	6130	62849	1447498
2015	1-10 Jun	2623062	2512418	129963	5265443

2016	11-20 Sep	376860	150144	2070968	2597972
2017	1-10 Oct	961222	18541	0	979763
2018	1-10 Oct	7181384	2936983	103852	10222219
2019	1-10 Apr	6422808	573218	24674	7020700
2020	21-30 Apr	4440093	5094082	703334	10237509
		40 cm depth			
2007	21-31 Jul	2868256	3098886	6495598	12462739
2008	11-31 Mar	1016938	1300662	1730405	4048005
2009	11-20 Jun	3876292	5058947	3101746	12036985
2010	11-20 Feb	1599263	115924	14214	1729401
2011	21-30 Nov	1358790	3238051	5176009	9772850
2012	11-20 Sep	2969274	4919427	3650411	11539112
2013	21-31 Aug	1347349	10987	10254	1368590
2014	1-10 Apr	1623873	38840	141646	1804359
2015	11-20 Aug	3154959	1690746	186978	5032683
2016	11-20 Sep	189872	74989	3308874	3573735
2017	11-20 Apr	578412	47378	1229186	1854975
2018	1-10 Jun	51665	0	2869286	2920951
2019	1-10 Apr	4369336	1323382	44952	5737670
2020	1-10 Jun	3762720	1633193	1241469	6637382
		100 cm depth			
2007	21-31 July	2443805	3389729	6485863	12319397
2008	1-10 Mar	1840206	936931	641133	3418270
2009	21-30 Jun	4188272	4874594	1709405	10772271
2010	21-31 Mar	1350737	253974	208416	1813128
2011	21-30 Nov	2718847	2154606	4545326	9418779
2012	1-10 Apr	2664729	2645592	5716684	11027005
2013	11-20 Mar	2475024	1538065	496961	4510050
2014	1-10 Apr	1001600	4425	66755	1072780
2015	11-20 Aug	2648589	1191357	215462	4055408
2016	11-20 Sep	106237	32853	4309522	4448612
2017	11-20 Oct	723452	44191	1086351	1853994
2018	1-10 Oct	0	0	3055429	3055429
2019	21-30 Apr	1812154	352121	16052	2180327
2020	1-10 Jun	3078626	1721219	983154	5782999
